# Assessment of the Shear Strength of Pile-to-Soil Interfaces Based on Pile Surface Topography Using Laser Scanning

**DOI:** 10.3390/s19051012

**Published:** 2019-02-27

**Authors:** Zbigniew Muszyński, Marek Wyjadłowski

**Affiliations:** 1 Faculty of Geoengineering, Mining and Geology, Wrocław University of Science and Technology, 50-370 Wrocław, Poland; zbigniew.muszynski@pwr.edu.pl; 2 Faculty of Civil Engineering, Wrocław University of Science and Technology, 50-370 Wrocław, Poland

**Keywords:** terrestrial laser scanning, LIDAR, shear strength, CFA piles, roughness parameters

## Abstract

This article presents in situ research on the side surface of continuous flight auger (CFA) foundation piles using a three-dimensional (3D) laser scanner (Leica ScanStation C10) in order to evaluate the morphology assessment of pile concrete surfaces in various geotechnical layers. Terrestrial laser scanning describes the 3D geometry of the construction with high spatial resolution and accuracy. A total of six areas were selected from the acquired point cloud for which a two-step approach for removing the form was applied. In the first step, the reference surface was fitted using the least squares method, and then, cylindrical projection of the surface was performed. In the second step, an operator of removal of the multi-plane form was applied. For each sample, height parameters (*Sq*, *Ssk*, *Sku*, *Sp*, *Sv*, *Sz*, *Sa*) and functional volume parameters (*Vmp*, *Vmc*, *Vvc*, *Vvv*) according to the standard ISO 25178-2:2012 were determined. Significant differences in the values of surface height and functional volume parameters were observed for each geotechnical layer where piles were formed. Because the piles remain embedded in the ground, in situ tests of the side surface of piles are rarely performed and taken into account in the assessment of pile bearing capacity. The study of surface topography is a crucial stage in the assessment of the shear strength at the interface between a concrete pile and the soil layer. The obtained concrete morphology assessments are applicable during the determination of the skin friction factor in the analytical or numerical estimation of pile shaft resistance. The proposed procedure of morphology evaluation may improve the fidelity of the assumed friction factor between the concrete and soil and increase the reliability of direct shear experiments.

## 1. Introduction

Topographic analysis of concrete surfaces is usually aimed at improving the quality or increasing the durability of concrete elements and structures. The protection of concrete against corrosion may be achieved through the use of various surface treatment technologies and the modification of surface parameters [1,2]. The appropriate preparation of concrete surfaces is also important for the achieved shear strength of structural layers connected with the concrete. Common to design codes is the qualitative evaluation of the surface roughness of the concrete substrate. The bond strength at the interface between the concrete and soil layers is important to ensure the monolithic behavior of composite members. The Eurocode 2 standard [3] classifies the roughness of a substrate surface as very smooth, smooth, and rough. This classification is clearly inaccurate, because it depends on a subjective assessment of the designer. Studies of the impact of the concrete surface treatment on its roughness and results of pull out tests are presented in [4,5,6]. In the case of geotechnical structures implemented in subsoil subbases, the roughness of the concrete surfaces affects the durability and strength of the structures. However, detailed studies of the surfaces of geotechnical structures are rarely performed, because access to underground objects is usually very limited. A few exceptions include excavated diaphragm walls or surfaces of concrete dams from the downstream side. Usually, in addition to non-destructive material tests [7], measurements of displacements using geodetic techniques [8,9] are performed in parallel.

**Figure 1:**
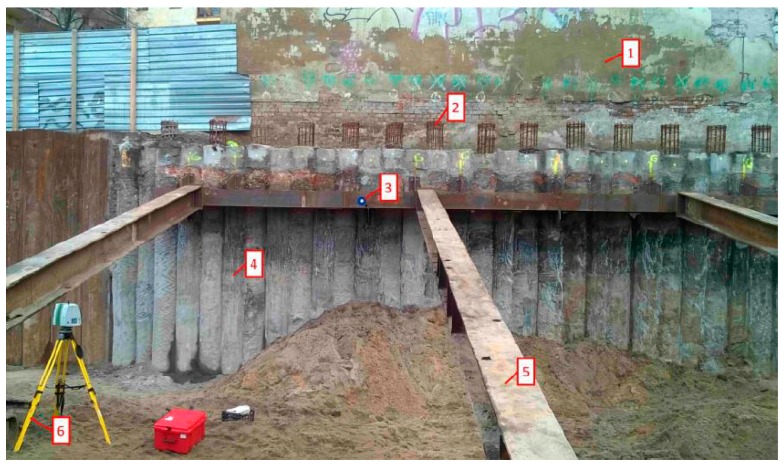
Protection of deep excavation by means of continuous flight auger (CFA) palisade: (**1**) the wall of the nearest existing building; (**2**) reinforcement cage; (**3**) special target used as the tie point for combining scanner positions; (**4**) palisade made of CFA piles; (**5**) steel strut; and (**6**) position of the laser scanner.

The technology of making continuous flight auger (CFA) piles affects the formation of the characteristic rough surface of the piles (Figure 1, detail 4). Piling is a cast, in situ process that is very suited to soft ground where deep casings or the use of drilling support fluids might otherwise be needed. The most significant distinction from conventional bored piles is that CFA piles do not create an open excavation. The sides of the hole are supported at all times by the soil-filled auger, eliminating the need for temporary casing or bentonite slurry. The construction sequence of a CFA pile is as follows:

- Drilling a full-length auger with a hollow stem (temporarily plugged) into the soil using a constant penetration rate;
- After reaching the design toe, concrete is pumped through the hollow stem of the auger while the rotating auger is extracted at the same time. It is important that the auger always remains embedded in the concrete and that a positive concrete pressure is maintained throughout the placement of the concrete;
- After completion of the concrete placement process, the reinforcement cage (Figure 1, detail 2) is thrown into fluid concrete.

Quality control is critical in the construction of CFA piles. CFA drilling rigs feature built-in devices to monitor the volume of the concrete pumped as the auger is extracted. The following parameters are usually recorded: penetration/uplift per revolution, auger depth, concrete supply per increment of auger uplift during placement, and injection pressure at the auger head. Concrete for deep foundations must have excellent workability criteria and sufficient resistance against bleeding to guarantee a non-defective end-product. The pressure effects from the hydraulic head and the self-weight of the concrete mass on the fresh concrete at the base of deep foundations cause the risk of bleeding and associated stability and quality issues [10].

In the case of concrete CFA piles, the roughness of their side surface also depends on the specific conditions of the ground base in which the piles were formed. At the same time, the surface parameters of the piles affect the shear strength between the concrete and the surrounding soil. The roughness of the pile shaft is of primary importance in the case of load transfer to the surrounding soil. The load transfer determines the stress state at the pile toe level and, consequently, the pile capacity and its settlement at the service load. Various aspects of this impact were analyzed by Baca [11]. Furthermore, combinations of loads in the case of steel tubular piles (where roughness and adhesion may be determined by rusting) were described by Rybak [12]. The coefficients of cohesion and friction are linked to the surface finishing treatment. In calculations of foundation piles, this phenomenon is very important for achieving bearing capacity by the pile’s side surface and in the scope of calculations of the palisade as a retaining structure.

The need for a detailed description of a pile’s side surface led to the dynamic development of methods and parameters for the quantitative description of surface irregularities. Currently, the principles of measurements and definitions of parameters that numerically describe 3D surface irregularities are normalized [13]. The number of used spatial parameters is constantly growing, and new parameters are emerging for the assessment of surfaces in various technologies and applications [1,14,15,16]. The most commonly used method of graphical presentation of the examined surface is an isometric image with appropriately selected dimensions and resolution.

In this work, the lateral surface of concrete piles forming a palisade, which secures an excavation, was analyzed. The deep excavation, which was located in an urbanized area, extended to 4.30 m below ground level. The piles were inserted in complex geotechnical conditions as secant with positive overlapping. The palisade was made of class C30/37 concrete. The piles were implemented with CFA technology in layered subsoil. The measurement of the geometric shape of the piles’ lateral surface, after their excavation, was performed with a laser scanner. After the appropriate treatment of the point cloud, six sub-areas were selected, and for these sub-areas, 3D models of the concrete surface were created and areal parameters describing the topography of the surface (height and functional volume) in accordance with the standard [13] were determined. The obtained results were associated with the geotechnical profile of the soil, which may be important in assessing the bearing capacity of the pile’s side surface.

## 2. Materials and Methods

### 2.1. Terrestrial Laser Scanning

Terrestrial laser scanning (TLS) is a dynamically developing measurement technology, which is extensively used in various fields of science and technology. Based on the method of measuring the distance to the examined object, laser scanners can be divided into three categories:

- Pulse scanners, in which the time of passage of the laser beam from the scanner to the object is measured (the so-called time-of-flight measurement);
- Phase scanners, in which the phase difference between the sent signal and the returned signal is measured (the so-called phase measurement technique); and
- Triangulation scanners, whose operation consists of detecting the position of a single laser beam reflected from the object and determining the distance to the measured object using trigonometric relations in a triangle.

Triangulation scanners are the most accurate laser scanners; however, they have a significant limitation regarding the distance range to the measured object, which usually does not exceed 1–2 m. However, there are uncommon models, which have a longer range of up to 50 m. In the case of more distant objects, phase and pulse scanners are usually applied. Pulse scanners have the longest range—up to several kilometers (e.g., Riegl VZ-6000)—although the measurement accuracy decreases considerably with the increasing distance. The effective range of a scanner determines its possible applications. Triangulation scanners are usually used in laboratory tests and metrological applications in the industry [17]. The roughness assessment of concrete surfaces in laboratory conditions using a triangulation scanner was described in [18,19], among others. Attempts to link parameters describing the topography of concrete surfaces (obtained on the basis of measurements with the use of a triangulation scanner) with concrete adhesion properties were presented in [4,5,6]. The scanning of larger and more complex objects (especially those that are in the process of construction) requires the use of laser scanners (phase or pulse scanners) and the performance of measurements from several positions. This usually requires the establishment of a precise geodetic control network and the measurement of special targets in order to combine the individual positions of the scanner. The process of combining point clouds from different scanner positions is called registration. There are various methods to register point clouds, a description of which may be found in [20,21], among others. In civil engineering, laser scanning is used for making inventories of concrete industrial heritage [22], precise building modeling [23], monitoring of tunnel deformation [24], and controlling the verticality of slender objects (e.g., industrial chimneys [25,26]), among other applications. The application of a pulse scanner to control the vertical displacement of a pile during a bi-directional static load test was presented in [27]. An important additional parameter recorded by pulse and phase scanners is the intensity of the laser beam reflection. In the case of terrestrial scanners, studies have been conducted regarding the use of the intensity parameter to recognize and classify the physical properties of concrete, sometimes using additional thermal imaging data [7]. The combination of TLS with thermal imaging to analyze the geometry of a reinforced cooling tower with a height of about 170 m was described in [28].

### 2.2. Principles of Roughness Parameters

Surface topography is informally understood to be a set of detailed three-dimensional (3D) features of a certain limited area of surface geometry. Part of the terminology associated with surface topography was taken from a two-dimensional description of roughness. In the case of a 3D description, the basic change is a mathematical determination of the surface by an equation of two variables, z(x,y). The area determined by this equation is a boundary that separates the object from another object, substance, or surface. For a two-dimensional (2D) description, this equation is only a default equation, and all the calculations come down to the analysis of the 2D cross-section. Only the combination of successive parallel cross-sections allows an approximate 3D image to be created. Therefore, the 2D measurements refer to the profile and are determined by measurements of profile irregularities, whereas 3D measurements refer directly to the surface and are described as measurements of topography or stereometric measurements. In order to conduct a comprehensive roughness analysis, around 60 3D roughness parameters were created to describe most of the surface morphology with regard to specific functions, properties, or applications [29]. In the last two decades, numerous attempts have been made to evaluate roughness parameters in 3D. In order to limit the increasing numbers of such parameters and to standardize their use, a standard [13] was developed [29]. The parameters are calculated on the measured surface without segmenting the surface into small sub-areas, which depend on the sampling length.

The 3D roughness parameters can be classified into the following groups [14]:

- Height parameters;
- Spatial parameters;
- Hybrid parameters;
- Functional parameters;
- Feature parameters; and
- Other 3D parameters.

However, the ISO standard [13] also distinguishes functional volume parameters.

For parameters and functions used in three-dimensional analysis, the letter “*S*” was assumed to be the equivalent denotation of the letter “*R*” in the profile parameters. The standard [13] defines symbols for surface texture parameters that have a prefix consisting of the capital letters *S* or *V* followed by one or several small letters that form a suffix. The prefix *S* is used for the majority of parameters (e.g., *Sq*, *Sdr*, *Smr*), with the alternative being volume parameters that start with the letter *V* (e.g., *Vmp*, *Vvc*). The most useful parameters for the evaluation of concrete surface morphology [30] and their definitions are presented in Table 1.

One of the important stages during the process of surface texture analysis is the removal of form. Metrological analysis is made on a flat surface, regardless of the original geometrical shape of the object under study. The surface of a real object after measurement is represented by a set of measurement data (e.g., cloud of points with known [*x*,*y*,*z*] coordinates from laser scanning), which constitutes the so-called extracted surface or primary surface (after optional filtering). Industrially manufactured objects have a certain original shape that is modified during further processing, which may be a plane, cylinder, cone, or sphere [31,32]. This original shape is called the nominal (base) form and should be removed from the extracted/primary surface before further metrological analysis. The form removal operation (the application of the so-called “F-Operator”) can be made by fitting with a nominal shape or filtering with an F nesting index [33]. Fitting with a nominal shape is usually performed using the least squares method. In the case of a very complicated surface, the fitting is performed using more sophisticated algorithms, such as polynomial fit or multi-plane fit. An example of such an F-Operator is the “remove multi-plane form” operator implemented in the MountainsMap Premium software v. 7.3 (Digital Surf). This operator removes general form and deformations, ignores transitional zones, separates slopes, and levels terraces and steps with preservation of their heights and morphology [31]. As a result, we obtain the so-called SF surface, for which the areal parameters can be calculated. If necessary, this surface can also be further filtered to separate long-scale components from short-scale components (waviness from roughness) before the additional step of the calculation of the areal parameters.

In this work, regarding CFA piles, we propose an original two-step approach for the application of an F-Operator. The first step involves removing the nominal form by means of fitting the cylinder using the least squares method and then unrolling the side surface of the cylinder (cylindrical projection). The obtained surface takes into account the real unevenness of the pile (e.g., zigzags). This surface contains complete information about the topography of the side surface of the pile (which is important in the estimation of the bearing capacity) and can be used for calculating the first set of areal parameters. Due to the large unevenness, which still remains on the analyzed surface and which may interfere with the reliability of some height parameters, we propose performing a second step of form removal using previously mentioned “remove multi-plane form” algorithm. For the surface prepared in this way, the chosen areal parameters are calculated again. This allows the topography of the side surface of the pile to be described while omitting the influence of the largest inequalities (e.g., zigzags).

### 2.3. Assessment of Shear Strength Parameters

According to the shear-friction theory, the load transfer mechanism of shear forces between two concrete layers consists of the following:

- Cohesion, due to mechanical interlocking between particles;
- Friction, due to the existence of compression stresses at the interface and to the relative displacement between concrete parts; and
- Dowel action, due to the deformation of the reinforcement bars crossing the interface.

The design shear stress $v_{Rdi}$ at the concrete–concrete interface is given in [3] by Equation (1):

(1) $$v_{Rdi}=c\cdot f_{ctd}+\mu \cdot \sigma_{n}+\rho \cdot f_{yd}\cdot \left(\mu \cdot \sin\alpha +\cos\alpha \right)$$

where *c*—cohesion—and *μ*—friction—are factors that depend on the roughness of the interface, $f_{ctd}$ is the design tensile strength of the concrete, $\sigma_{n}$ is the external normal stress acting on the interface, ρ is the reinforcement ratio, $f_{yd}$ is the yield strength of the reinforcement, and α is the angle between the shear reinforcement and the shear plane.

The roughness characteristics in the Eurocode 2 standard [3] are only descriptive and do not refer to the roughness parameters according to the standard [13]. With Equation (1) it is possible to describe the shear stress between the soil and the pile. The third component is omitted, and the design tensile strength of the concrete can be replaced by the strength of the soil.

Equation (1) can be used primarily when the piles are designed to strengthen the existing concrete geotechnical constructions, for example retaining structures. For piles operating as typical foundation piles, the estimation of load capacity according to the Eurocode 7 geotechnical guidelines [34] should be used. The analysis of the obtained scanning results can be carried out in relation to serviceability limit states (ULS) and limit state (LS). Pile load-carrying capacity (limit state) depends on various factors, including the following:

- Pile characteristics, such as pile length, cross section, and shape;
- Soil configuration and short- and long-term soil properties; and
- Pile installation method.

Piles resist applied loads through side friction (shaft or skin friction) and end-bearing. Friction piles resist a significant portion of their loads by the interface friction developed between their surface and the surrounding soils. The characteristic base resistance $R_{b;k}$ and shaft resistance $R_{s;k}$ may be determined directly from the ground parameters using the following Equations (2) and (3) given in [34]:

(2) $$R_{b;k}=A_{b}\cdot q_{b;k}$$

(3) $$R_{s;k}=\sum A_{s;i}\cdot q_{s;i;k}$$

where $A_{b}$ is base area, $A_{s;i}$ is surface area in the *i*-th layer, $q_{b;k}$ is characteristics of unit base resistance, and $q_{s;i;k}$ is characteristics of unit shaft resistance in the *i*-th layer.

The standard [34] gives Equation (3) for the shaft capacity; however, the problem is to determine the value $q_{s}$ of unit shaft resistance. Two widely used methods for pile design can be used to estimate the value of unit shaft resistance $q_{s;i;k}$:

- *α*—a method used to calculate the short-term load capacity (total stress) of piles in cohesive soils; and
- *β*—a method used to calculate the long-term load capacity (effective stress) of piles in both cohesive and non-cohesive soils.

The unit shaft resistance, $q_{s}$, between the pile and the surrounding soil is calculated in Method *β* according to Equation (4):

(4) $$q_{s}\left(z\right)=\mu \left(z\right)\cdot K\left(z\right)\cdot \sigma_{v}^{\prime }\left(z\right)=\tan\delta \left(z\right)\cdot K\left(z\right)\cdot \sigma_{v}^{\prime }\left(z\right)$$

where $\sigma_{v}^{\prime }\left(z\right)$ is the vertical effective stress, μ(z) is the interface friction, δ(z) is the pile skin friction angle, and K(z) is the rest pressure coefficient, which depends on the installation mode. Usually, K=K0 is defined by Equation (5):

(5) $$K0=\left(1-\sin\varphi^{\prime }\right)\cdot OCR^{0.5}\leq 3$$

where *φ*′ is effective friction angle and OCR is over-consolidation ratio.

These above factors are taken into account by Method *β* [35]. Niazi and Mayne [24] presented 15 methods for estimating pile unit shaft resistance within Method *β*. For bored in situ concrete piles, both qualitative and quantitative assumptions are made on the influence of interface friction. The most common assumption for a cohesive and non-cohesive soil is the tangent of 2/3 or 3/4 effective internal soil friction angle $\varphi^{\prime }$ as the angle δ of pile (wall) friction value for the soil–concrete interface. A shear test on the interface between coarse-grained soil and concrete was conducted by [36]. A simple shear test representing low stress conditions is obtainable for the concrete pile–soil interface and is more suitable for studying the shear displacement and deformation properties of the interface in a low stress state, while a torsional shear test simulation is more suitable for testing the strength of the interface [37].

### 2.4. Research Study Site

#### 2.4.1. General Description of Study Site

**Figure 2:**
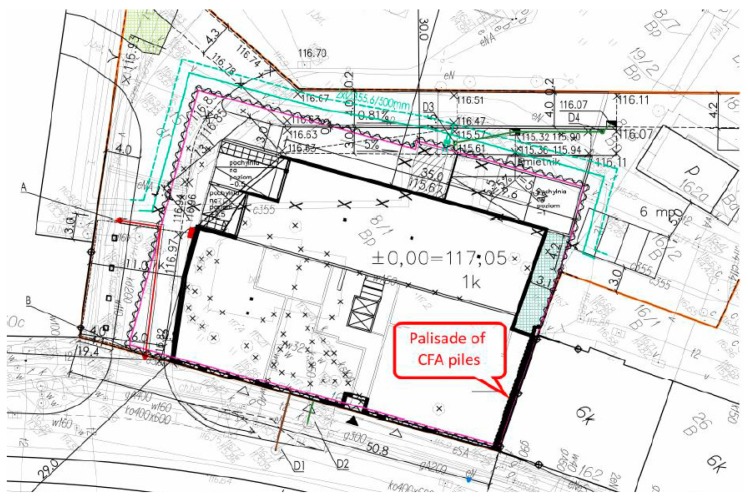
Map for design purposes showing the excavation range and location of the palisade.

A palisade made of CFA concrete piles was selected as a research object. This palisade was created as a retaining wall of a deep excavation located in an urbanized area (Figure 2). The original development of this area was compact and extended along the street. The building on the subject plot was demolished. The immediate vicinity included a building from the beginning of the 20th century adjacent to the plot’s border, which exhibited extensive wear and tear and poor technical condition. The commenced investment works consisted of developing the subject plot with a residential building with an underground garage. The palisade provided protection to a deep excavation along the plot’s border, which is adjacent to the gable wall of the neighboring building. The palisade consisted of 36 secant piles made using CFA technology [38] and drilled in a casing. The use of casing was adopted in order to limit the settlement of the neighboring building and the impact on the surroundings of the construction site. The length of protected gable wall was 15.30 m, the depth of the excavation during the examination reached 4.30 m below ground level, and the length and nominal diameter of the piles amounted to 10.70 m and 0.52 m, respectively. The piles were constructed as secant with an overlap of about 9–10 cm with each other. The palisade was made of class C30/37 concrete. Reinforcement profiles made of B500 class steel with a length of 8.0 m were installed in every other pile. Steel struts of the excavation were fixed to the palisade’s crown and the foundation slab at the bottom of the excavation. The piles were installed in August 2015, and the examination of the palisade in the excavation site was carried out in December 2015. The palisade was implemented in diverse geotechnical conditions. Geotechnical investigations showed the existence of subsurface layers of embankments and native soils (silty sands and medium sands in a medium-compacted state), as described in Table 2. The soil layers were determined and described according to the code detailed in [39].

**Figure 3:**
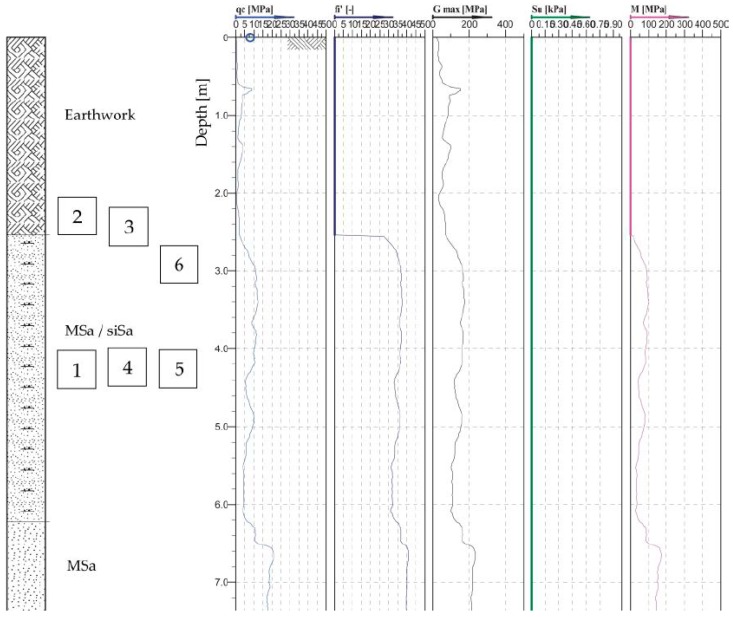
Geotechnical profile cross-section of the excavation site (from Cone Penetration Testing) with the location of the point cloud subsets.

A geotechnical cross-section of the excavation site is presented in Figure 3, where MSa is medium sand, siSa is silty sand, qc is cone tip resistance, fi’ is sleeve friction, G max is small strain shear modulus, Su is undrained shear strength, M is 1D compressibility, and I_D_ is density index.

Earthworks and uncovering of the palisade’s surface were carried out using manual equipment and partially mechanized equipment, which allowed the natural state of the concrete’s roughness to be maintained to a considerable extent.

#### 2.4.2. Acquisition and Processing of Data

**Figure 4:**
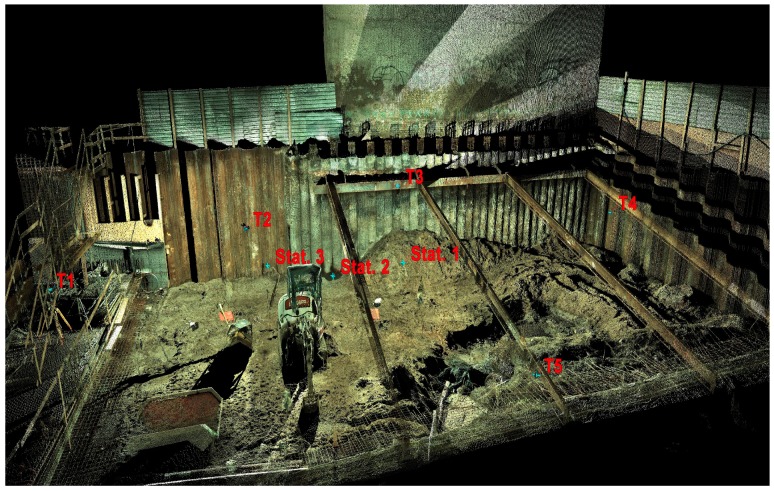
View of a combined point cloud colored on the basis of photos from the scanner with marked tie points (T1–T5) and scanner central points (Stat. 1–Stat. 3).

Laser scanning was carried out using a ScanStation C10 instrument (Leica Geosystems, AG). The measurement accuracy of the horizontal and vertical angles given by the manufacturer is 60 microradians, while the accuracy of the reflectorless rangefinder is 4 mm in the range up to 50 m. According to the manufacturer’s specifications, the accuracy of the surface modeling based on the acquired point cloud is 2 mm. The scanning of the palisade was carried out from three scanner positions in order to obtain favorable angles of incidence of the laser beam and ensure the overlapping of the point clouds. Figure 4 shows the central point of the scanner (the origin of the local scanner coordinate system) for all the scanner positions. Panoramic photos of the surroundings were taken at each position, and five measurement targets (tie points marked as T1–T5 in Figure 4) were measured, which allowed for the subsequent combination of point clouds (registration) in the Cyclone software v. 9.2.1 64-bit (Leica Geosystems, AG). The mean absolute error of registration (which represents the global accuracy of the point cloud transformations) calculated only on the basis of tie points signaled by targets was 0.7 mm. A view of the combined point cloud with the colors obtained from photos is presented in Figure 4.

## 3. Results

**Figure 5:**
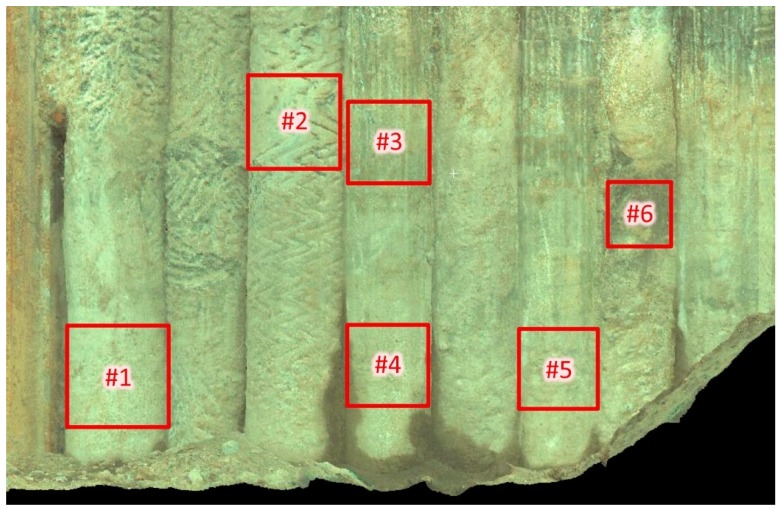
Location and numbering of the point cloud subsets on the CFA palisade.

**Figure 6:**
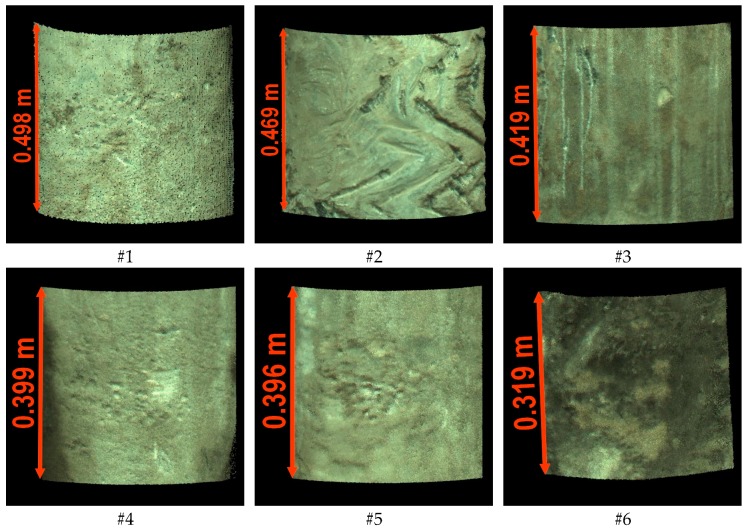
Subsets of the point cloud describing the surface of the piles.

**Figure 7:**
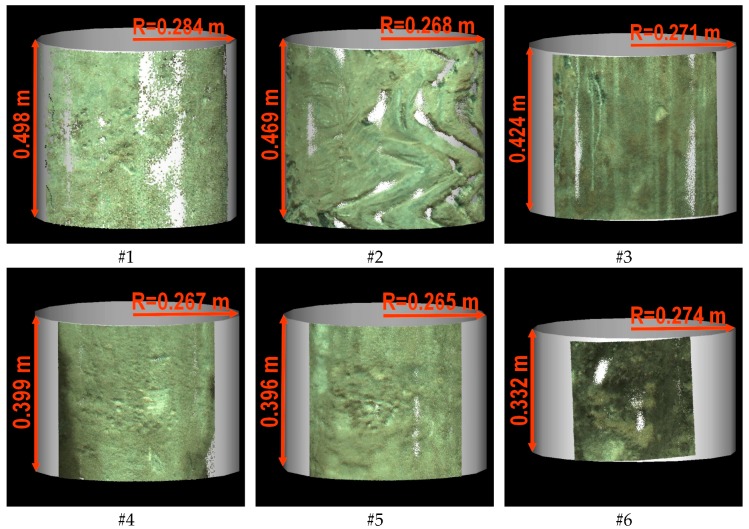
Cylinders fitted in the subsets of the point cloud using the least squares method.

For the purposes of subsequent works, six examination areas were selected from the scanned palisade, which are marked in Figure 5. For these areas, subsets of the point cloud limited by cubes with sides not exceeding 50 cm (the exact heights are given in Figure 6) were cut out from the cloud. Then, the first step of the form removal was performed. For each subset, the fitting of the cylinder into the point cloud using the least squares method (Figure 7) was carried out. Subsequently, separate cylindrical projections of each cloud subset were performed in the Mathcad 15.0 environment (Parametric Technology Corporation). From the obtained six surfaces, three samples (numbers 1–3) in the shape of a square with a side of 350 mm and three samples (numbers 4–6) in the shape of a square with a side of 200 mm were cut out in the CloudCompare software v. 2.9.1 64-bit (Open Source Project) [40]. The different size of the samples resulted from the desire to maintain a homogeneous soil for the entire surface of the sample. The radius of the fitted cylinder, as well as the quantity and density of points for each examination area, are summarized in Table 3.

### Determination of Roughness Parameters

Height parameters, which are summarized in Table 4, were calculated for all the samples using the MountainsMap Premium software v. 7.3 (Digital Surf), in accordance with the standard ISO 25178. Subsequently, the second step of the form removal was performed, and the samples were subjected to the process of removing a multi-plane form. Then, the height and the functional volume parameters were calculated (Table 5).

**Figure 8:**
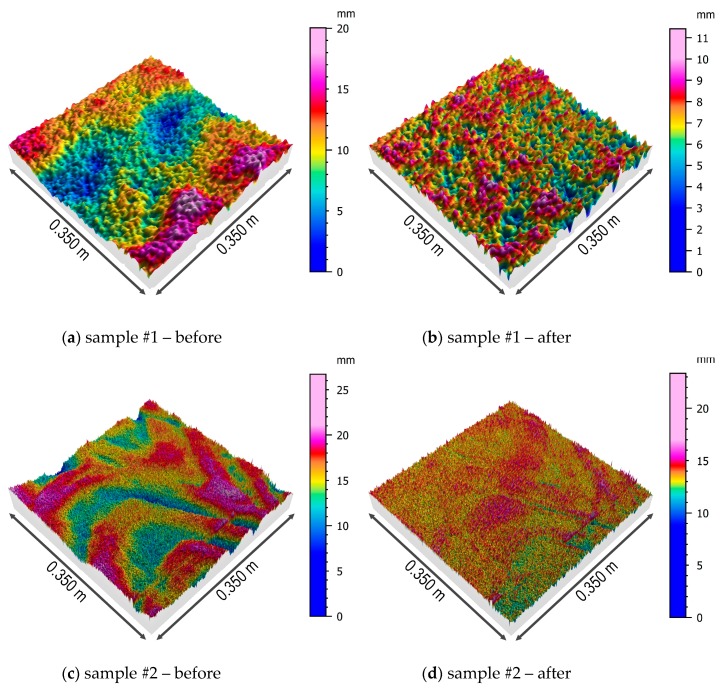

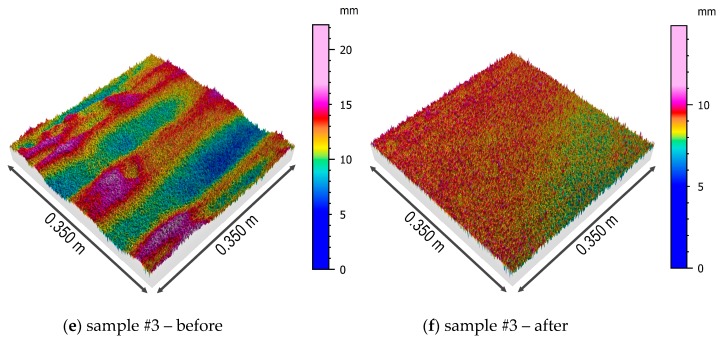
Isometric views of the surfaces of the samples #1, #2, and #3 before and after removing the multi-plane form.

**Figure 9:**
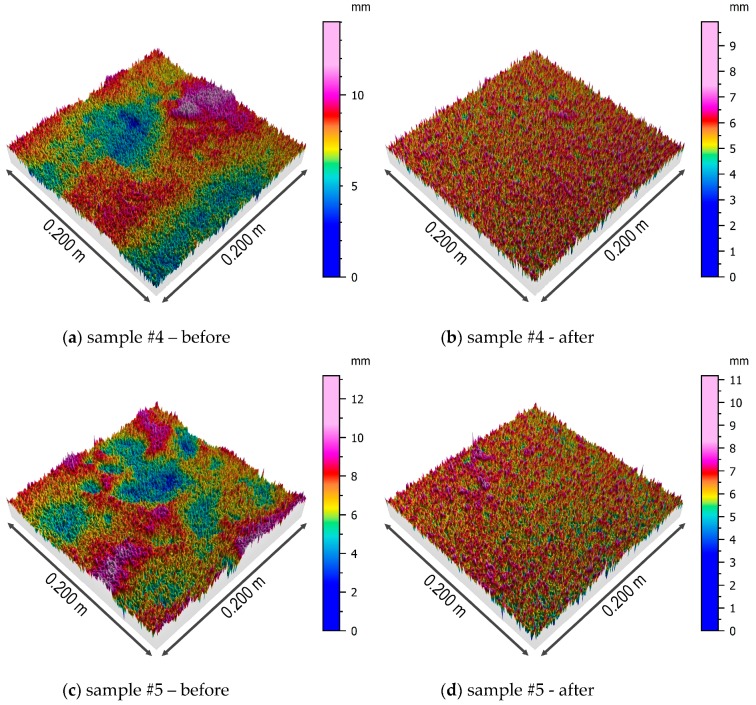

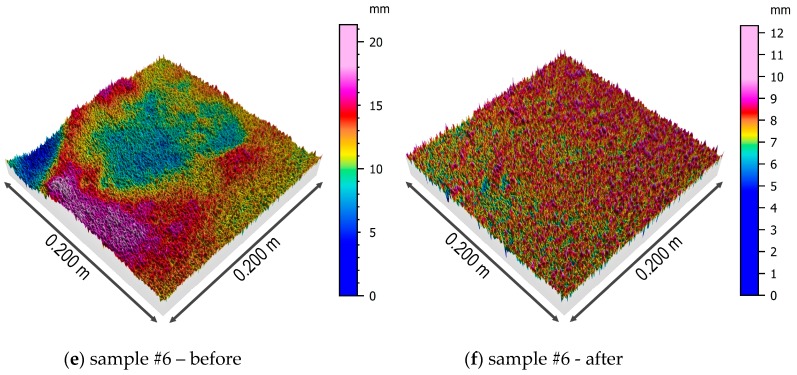
Isometric views of the surfaces of the samples #4, #5, and #6 before and after the removing multi-plane form.

Three-dimensional models of samples’ surfaces after the first step of the form removal and the surfaces of the samples obtained after the removal of the multi-plane form are presented in Figure 8 and Figure 9.

The first of the analyzed parameters of the concrete surface topography was the root-mean-square height of the surface, which is interpreted as the standard deviation of the height of the surface irregularities, i.e., the distance of the points from the reference surface. This parameter varies from 1.6 to 3.3 mm for the original model of the surface, with no clear differentiation in relation to the soil type. After removing the multi-plane form, the situation is similar, although the values of this parameter decrease due to understandable reasons (removing inequalities). The next analyzed parameter is the skewness coefficient, which is a measure of the distribution asymmetry. In the case of the analyzed samples, regardless of the type of soil in which the pile was formed, the distribution may be considered to be approximately symmetrical with a slight tendency to left-sided asymmetry. Kurtosis is a measure of the flattening of the distribution, and for a normal distribution amounts to 0. The results obtained for all the samples indicate the occurrence of leptokurtic distribution, more concentrated around the central value of the analyzed feature. It should be noted that the value of kurtosis increases in relation to all the samples after removing the multi-plane form. The arithmetic mean of the height is similar for all the samples and slightly decreases after removing the multi-plane form.

For the next three parameters, *Sp*, *Sv*, and *Sz*, a clear differentiation of values is visible depending on the type of soil in which the pile was formed. Samples 2, 3, and 6 came from places where the pile’s side surface was surrounded by earthwork, and for them, the abovementioned parameters reached higher values. This applies to the raw samples, as well as the samples after removing the multi-plane form. The remaining samples (1, 4, and 5), which came from places where the pile was formed in native ground, had lower values of the abovementioned three parameters of height (*Sp*, *Sv*, and *Sz*). This confirms a certain kind of engineering intuition that the type of soil surrounding the pile affects the shape and topography of its side surface. The values of shear strength for CFA piles in dense soils are greater than those for CFA piles in loose soils or earthwork [41].

As previously mentioned, the classification of surface roughness according to Eurocode 2 [3] is clearly inaccurate, because it depends on a subjective assessment of the engineer. A very smooth surface is considered to be a surface cast against steel, plastic, or specially prepared wooden molds. A smooth surface is a spliformed or extruded surface or a free surface left without further treatment after vibration. A rough surface is a surface that has at least 3 mm roughness at about 40 mm spacing, achieved by raking, exposing of aggregate, or other methods, giving an equivalent behavior. Based on the calculated parameters *Sp* and *Sv* and the definition of surface roughness in [3], the surface of the pile can be considered to be rough.

## 4. Discussion

TLS gives the possibility of a detailed description of the geometric shape of building structures, including in difficult field conditions prevailing at the construction site. Scanner positions were located inside the excavation, where typical construction works were in progress. For the issue considered in this work, the ideal solution would be using a triangulation scanner (due to their higher accuracy). However, this is usually not possible, due to the size of the measured object, excessive distance to the object, and winter conditions occurring at the construction site. Using a pulse scanner, with a distance to the object not exceeding 10 m, allowed a satisfactory measurement accuracy to be obtained.

The innovative application of TLS for topography description of the side surface of piles delivers valuable information. The foundation piles are rarely excavated, and there is no wider research describing the real roughness of the side surface of these piles in accordance with the standard ISO 25178-2:2012. Instead, qualitative (i.e., subjective and not quantitative) descriptions of roughness are used for pile design, based on laboratory tests, which are carried out on hypothetical samples that do not reflect the real shape of piles’ side surface. Real piles (as presented in this work) are usually more complex than the artificial samples prepared in the laboratory, due to the character of the surface of the palisade composed of secant piles in variable geotechnical conditions.

The change in soil strength parameters, grain size distribution, soil pressure, and, as a consequence, roughness parameters, occurs gradually in selected areas. The assessment and comparison of the obtained parameters of the concrete surface were carried out in relation to the results of the available research and roughness scale described in Eurocode 2. The lack of documented in situ tests of palisades in the literature justifies this procedure. Table 6 presents the proposed values for the cohesion and the coefficient of friction.

Similarly, if using the description of a rough surface according to [19], then a coefficient of friction μ=0.70 can be assumed for the examined surface of the pile, and this value can be used in Equation (1) when calculating the shear strength of the pile in connection with the concrete supporting structure. The occurrence of surface creasing reflecting the movement of the auger in area 2 before the removal of the multi-plane form can be interpreted as an “indented” type of surface with a high value of coefficient of friction, μ = 0.90. These are typical CFA pile surfaces.

The tests of concrete surface parameters described in [15] were carried out on the samples prepared in the laboratory. In these cases, the working conditions of the triangulation scanner were favorable in a controlled environment at a positive temperature and in the absence of other adverse atmospheric phenomena. Surface scanning in the laboratory is usually a preliminary step before further investigations, such as pull-outs [5,6], tension stresses, shear stresses, and a combination of shear and compression stresses [42] on samples of concrete surfaces with homogeneous, already known surface parameters. The research described in [42] was made with a profilometer and roughness parameters from the 2D group were obtained. The samples were subjected to further tests to determine the shear strength parameter. Maximum valley depth (*Rv*) presented an almost linear correlation with the concrete bond strength in shear. The results indicate that the *Rv* roughness parameter could be adequate to incorporate in design expression for the longitudinal shear strength of the interface between the concretes [42]. The current research on the CFA palisade is performed for 3D surface analysis, where the equivalent of the *Rv* parameter is the Dale void volume (*Vvv*) parameter. The *Vvv* parameter represents the void volume of dale at the areal material ratio *p*%. It can quantify the magnitude of the core surface, reduced peaks, and reduced valleys using the other functional volume parameters *Vmp*, *Vmc*, and *Vvc.* For sample areas 2 and 3, the values of the functional volume parameters *Vmp* and *Vvv* are higher than for samples 4 and 5. This can be ascribed to the particles of the composite earthwork layer with low strength parameters being more penetrated and moved by the concrete under forming pressure than the robust medium sand (MSa) soil. The peak material volume parameter *Vmp* describes the volume of concrete located on the highest peaks of the pile surface, which will be cracked or removed during the pile loading process. The volume featured by *Vvv* is filled with soil in the concrete valleys and again the values of the *Vvv* parameter are smaller for samples 4 and 5. Shaft friction between the concrete surface and the soil in the valleys is smaller, because the normal effective stress σ_z_ on the concrete surface in the cavities is less than the effective stress σ_z_ resulting from the soil rest pressure on the basic vertical surface of the pile. The obtained values of *Vmp* and *Vvv* explain the expected higher pile shaft resistance in the medium sand layer. Practical engineering application may take into account the influence of skewness and kurtosis on the static coefficient of friction. In [43], the effects of kurtosis and skewness on different levels of surface roughness were investigated independently. It was found that positive skewness values were associated with larger contact force, real area of contact, and tangential and adhesion forces than the Gaussian case, while negative skewness values predicted lower values tangential and adhesion forces and larger deviations from the Gaussian case. It was also found that distributions with kurtosis higher than 3 predict higher friction parameters compared with the Gaussian case, while distributions with kurtosis lower than 3 predict lower values than the Gaussian case [43]. In the case of the studied surface areas, lower shear strength can be expected for areas 2 and 3 than for areas 4 and 5.

The evaluation of the obtained roughness parameters is also of practical use for the issues of the numerical modeling of the structure–soil interaction. The interface between two different materials is of critical importance in numerical modeling. For instance, in the PLAXIS [44] finite element code, it is accounted for by providing special zero-thickness interface elements and suitable values of the strength reduction factor $R_{inter}$ [45] for implemented interface elements. The strength reduction factor $R_{inter}$ reduces the soil shear strength parameters *c* and *φ* into interface strength parameters $c_{i}$ and $\varphi_{i}$, according to the following equations:

(6) $$c_{i}=R_{inter}\cdot c$$

(7) $$\tan\varphi_{i}=R_{inter}\cdot \tan\varphi .$$

The normal stiffness and shear stiffness of the interface element are directly proportional to the soil stiffness. The strength reduction factor $R_{inter}$ depends on the roughness of the structure with respect to the surrounding soil. In a finite element model, interface elements are placed along the vertical limit surface between the pile and soil, and hence mobilized shear resistance along the interface can be modeled by adjusting the value of the $R_{inter}$ parameter. It is important to estimate a reasonably proper value of $R_{inter}$, as it significantly affects the results. Numerical examples show that the lower the interface value $R_{inter}$, the larger the bending moment in the excavation support [46]. The recommended values of the strength reduction factor $R_{inter}$ are presented in Table 7 [45].

The values listed in Table 7 do not directly take into account the type of surface roughness. Moormann [47] performed experiments with stiff clays and rough concrete surface typical of bored piles. For these arrangements, he found that there is no substantial reduction in either friction or cohesion. The estimation of the strength reduction factor can be made using a back-analysis calculation based on a full-scale field test and numerical model of the task. However, care must be taken when setting up a numerical model, as the combination of various assumptions (material model, soil stiffness parameters, mesh coarsening) may significantly influence the outcome of the numerical calculation. For a strength reduction factor for rough concrete surfaces, the upper limit of the range in Table 7 can be applied. On the basis of the tests for the investigated rough concrete surface and the proposed values in Table 7, it is possible to determine a value of $R_{inter}$ of around 0.70 in an earthwork layer and of around 1.0 in a medium sand layer.

According to [48], the pile skin friction angle δ in Equation (4) for concrete piles has a value of 2/3 or 3/4 of $\varphi^{\prime }$. In most arrangements of pile–soil interfaces, this assumption can lead to more conservative estimations of shear strength in the analytical method than in the numerical modeling of the task. Due to various estimates of pile shaft bearing capacity, it would be worthwhile to reproduce the substantial pile surface in the form of laboratory samples. Such concrete samples, as well as the ground conditions and pressure on the surface of the samples, can be modeled in direct shear apparatus or using a torsional test in order to determine the shear stress and skin friction using back-analysis. In [49], five diverse types of concrete sample were used to study the effect of surface shape on the stress displacement relationship; however, these samples did not map the real piles, and only the qualitative description of concrete roughness was applied.

The bearing capacity by the side surface for rough surfaces of the pile is undoubtedly greater than it is for smooth surfaces [49]. A mobilization of the shaft bearing capacity on the order of 1.5% of the pile diameter takes place with pile settlements, and a mobilization of the bearing capacity of the base with settlements on the order of 2–3% takes place [35,50]. The higher share of pile shaft bearing capacity in the total load capacity results in a more favorable characteristic of the load-settling curve. This is advantageous for the ULS state, with smaller settlements observed for the same load values.

## 5. Conclusions

The conducted studies allowed the formation of preliminary conclusions that are useful at the stage of determination of the pile’s carrying capacity:

- There are visible differences in the topography of the pile’s surface within various geotechnical layers;
- The method of performing geotechnical works and earthworks significantly affects the topography of the pile’s surface;
- The actual diameter of the pile in the layer of embankments and medium sand is greater than the nominal diameter of the casing;
- The obtained values of the roughness parameters of the CFA pile surface are greater than the analogical parameters for the surface of the sand-blasted concrete and shot-blasted concrete (i.e., after laboratory artificial treatment).

The consequence of the varied pile roughness parameters in the considered soil layers is the variable shear strength of the pile surface–soil interfaces in the layers under examination. The larger the roughness, the larger the axial strain, and the higher the pile skin friction.

Further studies of the surface roughness of foundation piles should be expanded by collecting concrete samples and conducting laboratory tests on the mechanical bond between the soil and the concrete surface. It would also be worthwhile to compare data obtained from photogrammetry, phase scanners, and pulse scanners in terms of the actually achieved accuracy of measurement. In terms of elaborating on the methodology, it is necessary to think about the usefulness of removing the multi-plane form (as the standard in [13] encourages) in the discussed applications. This procedure is aimed at eliminating residual surface irregularities (remaining after the removal of the curvature by means of the cylindrical projection) and focusing only on the roughness of the concrete. In the case of the assessment of the piles’ carrying capacity, large irregularities (in the shape of zigzags, see Figure 5) may be important. In this context, it seems justified to consider both sets of areal parameters (before and after removing the multi-plane form), as proposed in this work.

## Figures and Tables

**Table 1:** Examples of surface parameters and their definitions according to [13].

Name of Parameter	Definition
Height Parameters	
Root-mean-square height	$Sq=\sqrt{\frac{1}{A}\iint_{A}^{}{\left(Z\left(x,y\right)\right)}^{2}dxdy}$
Skewness	$Ssk=\frac{1}{Sq^{3}}\left[\frac{1}{A}\iint_{A}^{}{\left(Z\left(x,y\right)\right)}^{3}dxdy\right]$
Kurtosis	$Sku=1/S_{q}{}^{4}/A\iint_{A}^{}Z{\left(x,y\right)}^{4}dxdy$
Maximum peak height	$Sp=sup\left\{Z\left(x_{i},\;y_{i}\right)\right\}$
Maximum pit height	$Sv=\left|inf\left\{Z\left(x_{i},\;y_{i}\right)\right\}\right|$
Maximum height	Sz=Sp+Sv
Arithmetic mean height	$Sa=1/A\iint_{A}^{}\;\left|Z\left(x,y\right)\right|dxdy$
Functional Volume Parameters	
Peak material volume	Vmp=Vm(p)
Core material volume	Vmc=Vm(q)−Vm(p)
Core void volume	Vvc=Vv(p)−Vv(q)
Dale void volume	Vvv=Vv(q)

**Table 2:** Geotechnical description of the soil layer.

Subsets of Point Cloud	Soil Layer Description	Remarks
**#1, #4, #5**	MSa/siSa, I_D_ = 0.45	
**#3, #6**	Earthwork/MSa/siSa, I_D_ = 0.45	Transition from earthwork to MSa/siSa
**#2**	Earthwork (fill ground)	

**Table 3:** Parameters describing the subsets of the point cloud.

Subset of Point Cloud No.	#1	#2	#3	#4	#5	#6
Radius of fitted cylinder [m]	0.2842	0.2681	0.2709	0.2667	0.2650	0.2745
Number of points in subset	15,407	569,325	519,299	147,433	106,513	134,489
Density of points [pt/mm^2^]	0.13	4.65	4.24	3.69	2.66	3.36

**Table 4:** Results of the measurement of the three-dimensional (3D) roughness parameters for each sample before removing the multi-plane form.

Description	Name	Unit	Before Removing Multi-Plane Form—Sample No.					
			#1	#2	#3	#4	#5	#6
Root-mean-square height	*Sq*	mm	3.3	2.8	2.4	1.7	1.6	2.7
Skewness	*Ssk*	-	0.1	−0.4	−0.1	0.1	0.1	0.1
Kurtosis	*Sku*	-	2.7	3.0	2.6	3.0	3.0	3.0
Maximum peak height	*Sp*	mm	10.4	11.6	11.1	7.1	6.9	10.1
Maximum pit height	*Sv*	mm	9.6	15.1	11.1	7.0	6.3	11.2
Maximum height	*Sz*	mm	20.0	26.7	22.2	14.0	13.2	21.3
Arithmetic mean height	*Sa*	mm	2.7	2.3	1.9	1.3	1.3	2.1

**Table 5:** Results of the measurement of the 3D roughness parameters for each sample after removing multi-plane form.

Description	Name	Unit	After Removing Multi-Plane Form—Sample No.					
			#1	#2	#3	#4	#5	#6
Root-mean-square height	*Sq*	mm	1.2	1.3	1.0	0.8	0.9	0.9
Skewness	*Ssk*	-	−0.4	−0.2	−0.4	−0.1	−0.2	−0.1
Kurtosis	*Sku*	-	3.9	4.9	4.1	3.7	3.8	3.6
Maximum peak height	*Sp*	mm	4.5	10.3	6.4	4.8	5.3	5.0
Maximum pit height	*Sv*	mm	6.9	13.1	8.4	5.2	5.9	7.3
Maximum height	*Sz*	mm	11.4	23.3	14.8	9.9	11.2	12.3
Arithmetic mean height	*Sa*	mm	1.0	1.0	0.8	0.6	0.7	0.7
Peak material volume	*Vmp*	ml/m^2^	53.7	71.6	48.2	40.5	42.0	45.8
Core material volume	*Vmc*	ml/m^2^	1064	1046	888	704	752	788
Core void volume	*Vvc*	ml/m^2^	1388	1429	1165	949	1003	1061
Dale void volume	*Vvv*	ml/m^2^	166	176	143	101	110	112

**Table 6:** Coefficient of friction according the Eurocode 2 standard [3].

Type of Surface	Coefficient of Cohesion *c*	Coefficient of Friction *μ*
Very smooth	0.025–0.10	0.50
Smooth	0.20	0.60
Rough	0.40	0.70
Indented	0.50	0.90

**Table 7:** Suggested strength reduction factors ($R_{inter}$).

Soil–Structure Interaction	Strength Reduction Factor $R_{inter}$
Sand/steel	0.6–0.7
Clay/steel	0.5
Sand/concrete	1.0–0.8
Clay/concrete	1.0–0.7
Soil/geogrid (grouted body)	1.0
